# Are There Pysiological Affinities in the Blood

**Published:** 1883-01

**Authors:** Allen S. Heath

**Affiliations:** Professor of Cattle Practice Columbia Veterinary College, President of Farmers’ Club, etc.


### Art. II.—ARE THERE PHYSIOLOGICAL AFFINITIES IN THE BLOOD OF EACH BREED OF ANIMALS?

BY ALLEN S. HEATH, M.D.,

*Professor of Cattle Practice Columbia Veterinary College, President of Farmers' Club, etc.*

THE quality of blood has so much to do in the peculiar characteristics of the several breeds of animals, that I am at a loss otherwise to account for the potential influences of breed, unless there are active affinities in the blood.

The Holstein breed of cattle has its own peculiar physiological characteristics. The prominent features of this breed are: size, black and white color, large milk production, large digestive apparatus, short inturned horns, round bodies, and roundness of the bones of the limbs. These characters are so marked that even half-breeds, quarter-bloods, or those grades of only one-eighth blood, show these characters. How could this be but for the affinities for each other of the globules of Holstein blood?

Grades crossed upon grades of opposite characters show none of these affinities, nor predispositions.

Can the long breeding for a special purpose so impress the blood globules and the vital forces, that all of the minutest functions of the physical organisms shall be perpetuated in breeds, like copies of photographs by the artist, though the last likeness taken shall be a copy of the succeeding ones? These are reproductions of pictures with no variations; while animal reproductions must show that the originals have been modified by tints, shadings, and modifications of form and figure, however slight.

If at every dilution of blood, as in grades, no vital affinities be maintained in the blood, why the appearance of these characteristics even down to the tenth dilution on the "pure-bred," "thoroughbred" animals? There is no such thing as *pure blood* in domestic animals. The "thoroughbred" race-horse is only relatively pure-blooded. The dilution of cold blood has become extremely attenuated, and unlike the *homœopathic dilution*, it weakens at every dilution.

The consummation of the sexual act imports to the offspring of that union one individual of a dual character equally imported by each parent. This complex-animal essence—call it by whatsoever name you please—is possessed of a formitive power which develops two individualities in one. But for this, *pedigree* would be as meaningless as *prepotency*. Of course, I speak of the latter term as too often used to convey a false problem in the physiology of animal life. According to many advertisements, prepotency means that the advertised males possess the *impossible powers* to transmit their own characteristics exclusively without those of the female in any way interfering. If this is so, why use other agencies than the marvelously prepotent male? Prepotency rather means that affinity of blood, vitality of breed, which reproduces itself, its characters, its breed, from long-fixed characters, despite the divergent characteristics of the female, though modified by her.

If there are no accumulations of blood affinities when distant strains of blood reunite, why did the horses of the Messenger blood trot faster as Messenger blood accumulated in their veins? Or why did the accumulations of kindred blood tell in Bonnie Scotland and his descendants, though even of remote ancestry? It was the concentration, or accumulation, of Messenger blood in the Hambletonians and other trotters, that put *physical power* and *will power*, and smooth and rapid strides, that made the trotting of a mile possible in 2:10½ by the modern trotter.

Mr. Cole, of Solsville, Madison County, N. Y., purchased an imported cow in calf some twenty-eight or thirty years ago. The calf proved to be a male. These he in-bred with their descendants all this time. The breed was the Holderness. For a time the stock were red and white in color. Every generation grew darker red, until after a time the calves were born apparently black and white; but the red kept growing darker and darker until finally his valuable herd became permanently black and white. Let us realize the Holderness breed to see of what it is composed. The old red Durham, and the black and white Holstein furnished the blood. But Mr. Cole's original cow and calf, one or both, possessed a slight excess of Holstein blood, that took a long time to accumulate, so as to assert its characteristics. Not only has it done this in color, but it has also done it in form and milking qualities. The effect of the in and in-breeding is sure in firing the bones, and somewhat diminishing the size. The American Holderness has acquired such affinity of blood characteristics, that Mr. Cole deserves the credit of having originated a breed.

The American Shorthorns have been perfected and made superior to their foreign ancestors by the accumulations of the blood of the best strains of remote ancestry. There seems to be, not only affinities of consanguinity, but affinities of the refinements and of the superlative excellencies of breed strains as they commingle in modern breeding. This broad field of investigation has not yet been fully explored.

If my queries excite any useful thought and something instructive from some one proves more clear, I shall strive to profit by it in any future communication to the JOURNAL.

